# Vitamin D and calcium status in pre-eclampsia and pregnancy-induced hypertension

**DOI:** 10.17305/bb.2025.13081

**Published:** 2025-10-08

**Authors:** Wiktor Wojczakowski, Dominik Dłuski, Konrad Futyma

**Affiliations:** 1 Chair and Department of Obstetrics and Perinatology, Medical University of Lublin, Lublin, Poland; 2 Second Department of Gynecology, Medical University of Lublin, Lublin, Poland

**Keywords:** Vitamin D deficiency, calcium deficiency, hypertensive pregnancy

## Abstract

Hypertensive disorders of pregnancy are major causes of maternal and perinatal morbidity and mortality, and nutritional factors such as vitamin D and calcium have been proposed as modifiable risks; therefore, we investigated the association between maternal vitamin D and calcium status and pregnancy-induced hypertension (PIH) and pre-eclampsia (PE) and explored the relation with supplementation. In this observational cross-sectional study, 84 third-trimester women were enrolled from two hospitals in Lublin, Poland (41 PIH/PE, 43 controls). Serum total and ionised calcium, 25-hydroxyvitamin D [25(OH)D], and 1,25-dihydroxyvitamin D_3_ were measured using standardized immunoassays, and group differences, correlations, and multivariable logistic regression were applied with adjustment for body mass index (BMI), maternal age, gestational age, calcium fractions, and gestational diabetes. PIH/PE cases had lower 25(OH)D than controls (27.8 vs 35.7 ng/mL; *P* ═ 0.012) and higher BMI (33.0 vs 27.5 kg/m^2^; *P* < 0.001), while total and ionised calcium and 1,25-dihydroxyvitamin D_3_ were similar (all *P* ≥ 0.40); supplement use was more frequent among controls (84% vs 73%). In adjusted models, higher BMI increased the odds of PIH/PE (OR 1.19 per kg/m^2^) and higher 25(OH)D was protective (OR 0.92 per ng/mL); discrimination was fair (area under the curve 0.78). These findings support an association between vitamin D insufficiency and obesity with hypertensive pregnancy disorders and suggest preserved calcium homeostasis, but given the cross-sectional design, third-trimester sampling, small sample size, and non-standardised supplementation, causal inference and preventive recommendations cannot be made; larger prospective studies beginning in early pregnancy are warranted to test whether optimising vitamin D and calcium can reduce hypertensive complications.

## Introduction

Clinical descriptions of eclampsia date back over 2400 years, while pre-eclampsia (PE)—previously termed “toxicaemia of pregnancy”—was recognized as a distinct clinical syndrome only in the 19th century [1]. Despite its historical recognition, the pathophysiology of PE remains incompletely understood, which hinders the development of effective treatment options [1]. 

Pregnancy-induced hypertension (PIH) affects 3.6% to 9.1% of pregnancies, with PE impacting 1.4% to 4.0% of pregnant women. Collectively, these conditions contribute significantly to maternal and perinatal morbidity and mortality, with PE responsible for over 70,000 maternal deaths and 500,000 fetal deaths annually worldwide [2]. Although eclampsia is less common, it accounts for approximately 4.2% of maternal deaths and up to 30% of neonatal deaths [3].

PE is linked to various health problems in offspring [4], including a 1.3-fold increased risk of stillbirth and a 2-fold increase in the risk of neonatal death [5]. It is also a major contributor to fetal growth restriction (FGR), leading to infants being small for their gestational age in approximately 20% of full-term births and 60% of premature births [6]. Moreover, PE is a significant cause of prematurity; a recent large multicenter study found that the proportion of premature infants among women with PE is 36% [7], compared to approximately 10% in the general population [8]. Kajantie et al. [9] demonstrated an almost 2-fold increase in the risk of stroke among individuals whose mothers experienced PE.

In addition to mortality, the long-term consequences for mothers are notable. A meta-analysis published in 2007, which included around 200,000 cases of PE, revealed relative risks of 3.7, 2.16, and 1.81 for hypertension, ischemic heart disease, and stroke, respectively, after a mean follow-up of 10–15 years [10]. Women with early-onset PE exhibited an increased risk of cardiovascular disease (CVD) risk factors, such as elevated fasting glucose, insulin, triglycerides, and total cholesterol, in comparison to those with late-onset PE or PIH [11].

Women with a history of PE are at a heightened risk of developing diabetes, even in the absence of gestational diabetes [12]. They also face an increased likelihood of chronic kidney disease and hypertensive kidney disease, with a fivefold greater risk of developing end-stage renal disease compared to those without PE [13]. Additionally, there is a higher risk of neurological disorders, including a threefold increase in the likelihood of vascular dementia and potentially increased susceptibility to disorders affecting perception, memory, and motor function [14].

Nutritional factors, such as calcium and vitamin D status, have emerged as potentially modifiable risk factors. Up to 60% of women exhibit insufficient blood levels of vitamin D (below 30 ng/mL) and calcium (below 9 mg/dL) [15]. Multiple randomized controlled trials and meta-analyses indicate that vitamin D supplementation during pregnancy can reduce the risk of PE by approximately 40%–60% [16–19]. Likewise, calcium supplementation (≥1 g/day) can decrease the risk of PE by about 50%, with both low- and high-dose regimens being effective, particularly among women with low baseline calcium intake [20–22]. Despite the extensive literature, the challenge remains in determining the optimal vitamin D concentrations that would effectively reduce the risk of developing PE [23].

## Materials and methods

This observational study primarily aimed to determine the association between serum levels of vitamin D and calcium and the development of PIH and PE in pregnant women. A secondary objective was to evaluate the effect of vitamin D and calcium supplementation on mitigating the risk of these conditions.

The study population consisted of women referred to the obstetric clinic in two hospitals in Lublin, who were receiving antenatal care. Women willing to participate were asked to sign consent forms.

A total of 84 patients in the third trimester of pregnancy were recruited: 41 with PIH/PE (research group) and 43 healthy pregnant women (control group).

Inclusion criteria for the study included singleton pregnancies, maternal age between 18 and 45 years, and diagnosis of PIH or PE after 30 weeks of gestation, with blood pressure readings ≥140/90 mmHg. All participants provided written informed consent to participate in the study.

Exclusion criteria included women who declined to participate, as well as those with life-threatening conditions that would prevent study participation, such as eclampsia, renal failure, or other severe comorbidities. Pregnancies resulting from *in vitro* fertilization (IVF) were also excluded.

The control group comprised singleton healthy pregnant women with no history of hypertension, gestational diabetes, or other pregnancy complications. A standardized questionnaire was employed to characterize participants, including gravidity, age, height, current weight, body mass index (BMI), and gestational age. Each participant in the third trimester had blood drawn for analysis of vitamin D, its inactive form 25(OH)D, its active form 1,25(OH)_2_D_3_, and calcium levels.

Measurements of serum 25(OH)D, 1,25(OH)_2_D_3_, and calcium were conducted using the LIAISON^®^ XL analyzer (DiaSorin, Saluggia, Italy). Calibration was performed according to the manufacturer’s instructions using certified reference materials. The lower limits of detection were 2 ng/mL for 25(OH)D and 5 pg/mL for 1,25(OH)_2_D_3_. Internal quality control was conducted daily at two concentration levels, yielding coefficients of variation of 6.2% for 25(OH)D and 7.8% for 1,25(OH)_2_D_3_. The laboratory also participated in the national external quality assessment program (Polish Centre for Accreditation/national proficiency testing scheme), with all external quality control results within acceptable limits.

The study received approval from the Bioethics Committee at the Medical University of Lublin, under Bioethics Committee Resolution No. KE-0254/248/12/2022 and Resolution No. KE-0254/95/05/2024. Concentration determinations were performed by the hospital laboratories of the University Clinical Hospital No. 4 in Lublin and the Regional Specialist Hospital in Lublin.

Correlation analysis was conducted to assess relationships between serum vitamin D concentrations and other continuous variables, including ionized calcium and BMI. The selection of the correlation method—Pearson’s product-moment correlation or Spearman’s rank-order correlation—was based on the distribution profiles of the variables.

All continuous demographic and biochemical variables were expressed as mean ± standard deviation (SD). Normality of distributions was assessed using the Shapiro–Wilk test and Q–Q plots, while the equality of variances was evaluated using Levene’s test. For group comparisons, the Student’s *t*-test was employed for normally distributed data (reporting *t* and degrees of freedom), while the Mann–Whitney *U* test was applied for non-parametric data (reporting U). Correlations were analyzed using Pearson’s *r* for normally distributed variables and Spearman’s rho for non-normal variables, with all coefficients reported alongside 95% confidence intervals (CIs). To ensure transparency, all statistical test results, including those initially deemed non-significant, are reported with exact *P* values and 95% CIs for mean differences. In instances where multiple biomarkers were tested, the Benjamini–Hochberg false discovery rate (FDR) correction was applied, and both raw and adjusted *P* values are presented. Independent risk factors for the development of hypertensive disorders of pregnancy were assessed using multivariable logistic regression. Given the number of biomarkers tested and the relatively small sample size, formal corrections for multiple comparisons (e.g., FDR) were not applied; instead, the analyses are framed as exploratory, emphasizing effect sizes and clinical plausibility over isolated *P* values.

A post-hoc power calculation was performed for the two main comparisons (BMI and 25(OH)D levels between cases and controls) using a two-sample *t*-test (α ═ 0.05, two-sided). The observed effect size (Cohen’s d) for BMI was 1.21, yielding a post-hoc power of 99.9%. For 25(OH)D, Cohen’s d was 0.76, corresponding to a power of 74%. These results indicate that the study was adequately powered to detect differences in BMI and moderately powered for differences in 25(OH)D.

This study was observational in design, and its reporting adheres to the Strengthening the Reporting of Observational Studies in Epidemiology (STROBE) guidelines. The study followed the Generative Artificial Intelligence in Medical Research (GAMER) framework for transparent reporting of AI-assisted analyses. Generative AI tools were employed to support the structuring of statistical queries, validation of numerical consistency, table formatting, and biochemical unit conversions. Specifically, ChatGPT (OpenAI, GPT-5, September 2025 release) and Gemini Advanced (Google, 1.5 Pro, August 2025 update) were used in accordance with GAMER guidelines. These tools served solely as analytical and editorial assistants and were not involved in primary data analysis or statistical modeling. All statistical calculations were independently conducted and re-verified using standard statistical software (R 4.3.3 and SPSS 29). Full responsibility for data integrity, accuracy of analyses, and clinical interpretations rests solely with the authors.

**Table 1:** Participant characteristics

**Characteristic**	**Research group (*n* ═ 41)**	**Control group (*n* ═ 43)**
Mean age (Years)	31.5	30.1
Mean BMI (kg/m^2^)	32.96 ± 5.18	27.52 ± 3.77
Mean gestational age (weeks)	34.9 ± 3.3	37.2 ± 3.3
Pregnancy count - % distribution	I-51.22%, II-17.07%, III-9.76%, IV-9.76%, V-9.76%, VII-2.43%	I-55.81%, II-32.56, III-9.3%, V-2.33%
Vitamin D_3_ supplementation	73.2%	83.7%
Diet modification reported	43.9%	11.6%
Most frequent comorbidities	GDM 28%, Hypothyroidism 26%, Pre-eclampsia 7%	N/A

Note: Values are presented as means ± standard deviations. Research Group: Patients with diagnosed hypertension; Control group: Patients without recorded comorbidities; Supplementation: Includes any prenatal vitamins or mineral intake; Pregnancy count indicates the number of pregnancies a participant has experienced, with percentages shown for each occurrence (e.g., first, second, and third). Diet modification: Vegetarian, gluten-free, diabetic, low glycaemic index, or light diets. Comorbidities: Only non-hypertensive conditions reported; Abbreviations: BMI: Body mass index; D_3_: Vitamin D3 (cholecalciferol); GDM: Gestational diabetes mellitus; N/A: Not applicable.

### Ethical statement

This study was approved by the Bioethics Committee of the Medical University of Lublin (Resolutions No. KE-0254/248/12/2022 and KE-0254/95/05/2024). All participants provided written informed consent prior to inclusion in the study. The study was conducted in accordance with the principles of the Declaration of Helsinki.

### Statistical analysis

Statistical analyses were performed using Statistica v13.3 (StatSoft Inc., Tulsa, OK, USA). Continuous variables are presented as mean ± SD. Data distribution was assessed using the Shapiro–Wilk test, supported by inspection of Q–Q plots. For normally distributed variables, group comparisons were performed using the independent samples Student’s *t*-test; for non-normally distributed variables, the Mann–Whitney *U* test was employed. Categorical variables were analyzed using the χ^2^ test or Fisher’s exact test, as appropriate. A two-tailed significance level of α ═ 0.05 was adopted, with exact *P* values and 95% CIs reported. Given the number of biomarkers analyzed and the relatively small sample size, no formal correction for multiple testing was applied; instead, analyses are presented as exploratory, focusing on effect sizes and clinical relevance.

## Results

A total of 84 participants met the inclusion criteria for this study, which comprised two cohorts: those with PIH/PE (PIH/PE/research group, *n* ═ 41) and normotensive controls (*n* ═ 43). All participants had singleton pregnancies conceived naturally. Within the PIH/PE group, three participants had confirmed PE; due to the small sample size, instances of PIH and PE were aggregated for the primary analysis. The demographic and clinical characteristics of both groups are summarized in Table 1.

Among participants with PIH, 73.2% reported appropriate supplementation, and 43.9% adhered to dietary guidelines. In contrast, 83.7% of healthy controls utilized supplementation, while only 11.6% followed dietary recommendations. These findings indicate that, although supplementation is relatively common in both groups, adherence to dietary guidelines is significantly lower among those with PIH/PE.

The mean maternal age was 31.5 ± 5.1 years for the PIH/PE group and 30.1 ± 5.3 years for the control group (*P* ═ 0.22, 95% CI for mean difference –0.88 to 3.97). A statistically significant difference was noted in mean gestational age (GA) at the time of sample collection: the study group had a mean GA of 34.9 ± 3.3 weeks, while the control group had a mean GA of 37.2 ± 3.3 weeks (*P* ═ 0.0022, 95% CI –3.70 to –0.74). This disparity reflects the nature of the condition, as patients with PIH/PE often deliver earlier. The BMI was significantly higher in the PIH/PE group (32.96 ± 5.18 kg/m^2^) compared to the control group (27.52 ± 3.77 kg/m^2^, *P* < 0.001, 95% CI 3.05–7.83). Additionally, 12 women in the PIH/PE group had gestational diabetes, compared to one in the control group.

Table 2 presents the serum concentrations of various biochemical parameters analyzed in both study groups, including total calcium, ionized calcium, 25-hydroxyvitamin D (25(OH)D), and the active form of vitamin D_3_ (1,25(OH)_2_D_3_). Statistical significance was assessed using appropriate parametric or non-parametric methods where applicable.

**Table 2:** Serum parameter concentrations

**Parameter**	**Research group**	**Control group**	***P* value**	**Interpretation**
Total calcium (mg/dL)	9.14 ± 0.42	9.14 ± 0.39	0.98	No significant difference
Ionised calcium (mmol/L)	1.214 ± 0.070	1.205 ± 0.042	0.58	No significant difference
Total 25(OH)D (ng/mL)	27.8 ± 10.2	35.7 ± 10.8	0.0118	**Statistically and clinically significant difference**
Vitamin D_3_ (1,25(OH)_2_D_3_) (pg/mL)	126.2 ± 40.3	136.4 ± 51.6	0.4	No significant difference

Note: Values of total calcium, ionized calcium, vitamin D_3_ (1,25(OH)2D_3_), and total vitamin D (25(OH)D) are presented as means ± standard deviations for patients with pregnancy-induced hypertension (PIH) and healthy controls. Results of Student’s *t*-test are presented along with significance levels. In healthy persons, levels equal 25 to 40 ng/mL (62.4 to 99.8 nmol/L) for 25(OH) D_3_ and 19,9  79,3 pg/mL for 1,25(OH)2D_3_. The normal serum total calcium concentration in an adult is 8.6 10.2 mg/dL, ionised calcium 1.15–1.32 mmol/L.

The concentration of 25-hydroxyvitamin D revealed a statistically significant difference between the groups. The mean concentration in the PIH/PE group was 27.8 ± 10.2 ng/mL, categorizing this group as insufficient (below 30 ng/mL, equivalent to 75 nmol/L). In contrast, the control group had a mean concentration of 35.7 ± 10.8 ng/mL, with this difference being statistically significant (*P* ═ 0.0118, mean difference –7.9 ng/mL, 95% CI –14.0 to –1.8).

The mean concentrations of the active form of vitamin D did not differ significantly: 126.2 ± 40.3 pg/mL in the PIH/PE group vs 136.4 ± 51.6 pg/mL in the control group (*P* ═ 0.40, 95% CI –35.0 to 14.0). Both values are substantially elevated compared to the reference range for non-pregnant individuals (19.9–79.3 pg/mL), reflecting the physiological increase in renal and placental 1α-hydroxylase activity during the third trimester of pregnancy.

Neither total calcium (9.14 ± 0.42 vs 9.14 ± 0.39 mg/dL, *P* ═ 0.98, 95% CI –0.19 to 0.18) nor ionized calcium (1.214 ± 0.070 vs 1.205 ± 0.042 mmol/L, *P* ═ 0.58, 95% CI –0.023 to 0.041) demonstrated statistically significant differences. These results suggest effective calcium homeostatic mechanisms that maintain calcium concentration within a narrow range, even at the expense of systemic reserves.

**Figure 1.**
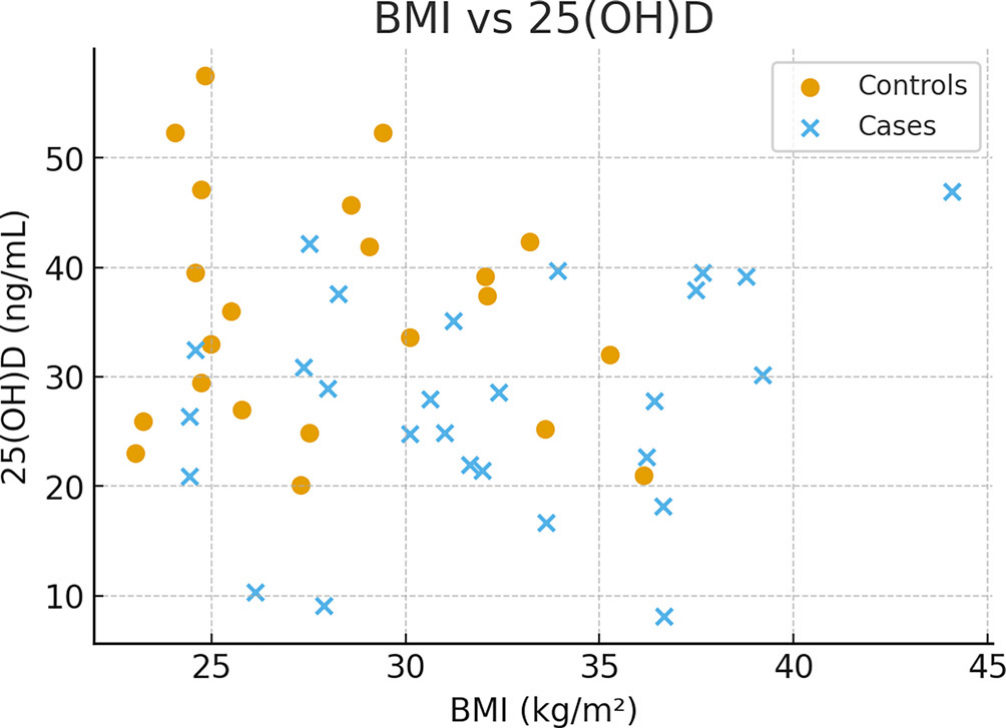
**Scatter plot showing the relationship between maternal body mass index (BMI, kg/m^2^) and serum 25-hydroxyvitamin D [25(OH)D] concentration (ng/mL).** Each point represents one participant; the solid line indicates the regression fit with 95% confidence interval (shaded area). Abbreviation: BMI: Body mass index.

**Figure 2.**
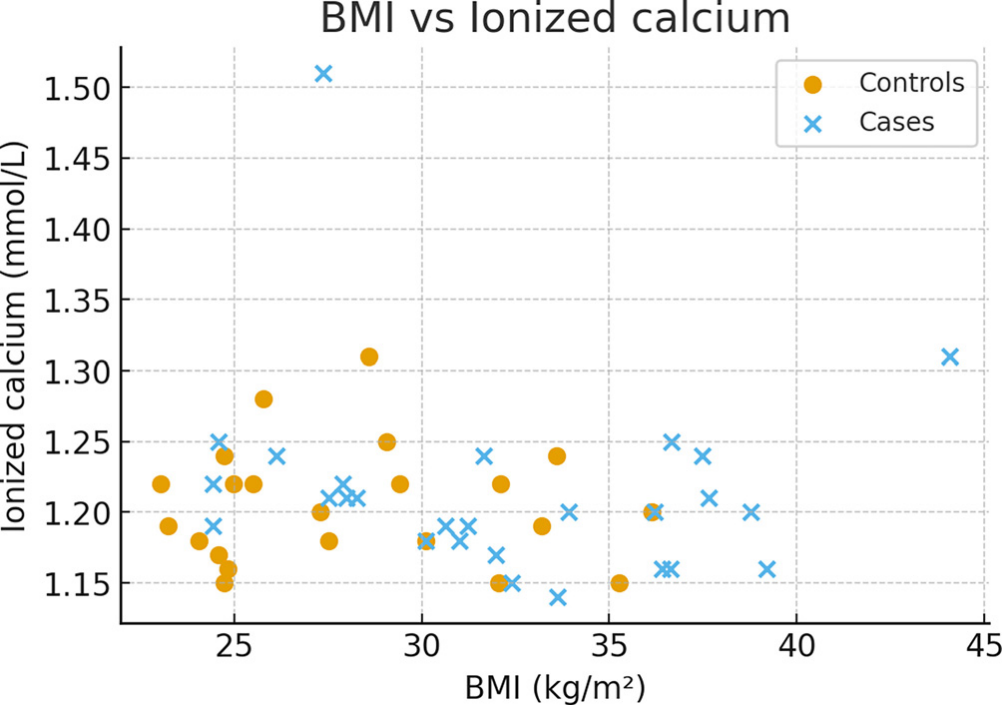
**Scatter plot showing the relationship between maternal body mass index (BMI, kg/m^2^) and serum ionised calcium concentration (mmol/L).** Each point represents one participant; the solid line indicates the regression fit with 95% confidence interval (shaded area). Abbreviation: BMI: Body mass index.

Scatter plots depicting the relationship between BMI and 25(OH)D, as well as BMI and ionized calcium, are provided in Figures 1 and 2.

The correlation coefficients are as follows:

BMI and 25(OH)D: Spearman *r* ═ –0.04 (95% CI –0.32 to 0.25), *P* ═ 0.76.

BMI and ionized calcium: Pearson *r* ═ –0.11 (95% CI –0.39 to 0.19), *P* ═ 0.47.

25(OH)D and 1,25(OH)_2_D_3_: Spearman *r* ═ 0.16 (95% CI –0.13 to 0.42), *P* ═ 0.29.

No correlations remained significant after applying the Benjamini–Hochberg correction for multiple testing (FDR-adjusted *P* > 0.1).

A multivariable logistic regression model was constructed to evaluate independent predictors of PIH/PE. After imputing missing values, the final model included BMI, serum 25(OH)D, maternal age, gestational age, total calcium, ionized calcium, and gestational diabetes (Table 3). In the adjusted analysis, BMI was identified as a significant risk factor (OR = 1.19 per 1 kg/m^2^ increase; 95% CI 1.02–1.38; *P* ═ 0.023), while higher concentrations of 25(OH)D were protective (OR = 0.92 per 1 ng/mL increase; 95% CI 0.85–0.997; *P* ═ 0.042). Gestational diabetes exhibited a non-significant trend toward increased risk, whereas maternal age, gestational age, total calcium, and ionized calcium did not achieve statistical significance (all *P* > 0.2). Model calibration was acceptable (Hosmer–Lemeshow test, *P* ═ 0.62). The discriminative ability of the model was fair, with an area under the curve (AUC) of 0.78 (95% CI 0.67–0.88) and a Brier score of 0.19.

## Discussion

This study aimed to investigate the association between serum concentrations of total calcium, ionized calcium, 25-hydroxyvitamin D (25(OH)D), and the active form of vitamin D_3_ (1,25(OH)_2_D_3_) in relation to PIH and PE. The primary finding reveals that women with PIH/PE exhibited significantly lower serum 25(OH)D concentrations and higher BMI compared to healthy controls, while calcium and 1,25(OH)_2_D_3_ levels did not show significant differences. These results support the hypothesis that disturbances in calcium–phosphate metabolism and vitamin D insufficiency may contribute to the pathophysiology of hypertensive disorders during pregnancy [24, 25].

**Table 3:** Multivariable logistic regression analysis for predictors of pregnancy-induced hypertension

**Variable**	**OR**	**95% CI**	***P* value**
BMI (kg/m^2^)	1.19	1.02–1.38	**0.023**
25(OH)D (ng/mL)	0.92	0.85–1.00	**0.042**
Gestational week	0.90	0.78–1.03	0.118

Note: Results of multivariable logistic regression identifying independent predictors of pregnancy-induced hypertension (including pre-eclampsia). The model included maternal BMI, serum 25(OH)D concentration, gestational week. Statistically significant predictors were higher BMI, lower 25(OH)D concentration. Odds ratios (OR) are presented with 95% confidence intervals (CI) and *P* values. Abbreviation: BMI: Body mass index.

The mean serum 25(OH)D levels demonstrated a notable statistical and clinical difference. Patients in the study group had significantly lower total vitamin D levels than healthy individuals, potentially increasing their risk for diseases associated with vitamin D deficiency.

The active form of vitamin D (1,25(OH)_2_D_3_) regulates the transcription and function of genes essential for normal implantation, placental invasion, and angiogenesis. The observed differences in concentrations (126.2 vs 136.4) were minimal and likely attributable to the limited sample size. Nevertheless, monitoring vitamin D levels in patients with pregnancy complications is critical due to its significant role in calcium metabolism and bone health.

Since blood samples were primarily collected in the third trimester following clinical diagnosis, our data should be interpreted as cross-sectional associations rather than evidence of causality or predictive capability. The observed lower levels of 25(OH)D in women with PIH/PE warrant further prospective investigation during early pregnancy before any screening or preventive recommendations can be made.

Vitamin D, via the activation of the vitamin D receptor (VDR), modulates the expression of genes that regulate cardiovascular system function, angiogenesis, and trophoblast implantation processes. Deficiencies in vitamin D may lead to endothelial dysfunction, increased oxidative stress, and activation of the renin–angiotensin–aldosterone system (RAA), which contributes to increased vascular resistance and elevated blood pressure [26–28]. It is proposed that vitamin D influences blood pressure through the regulation of endothelial function [29].

The data revealed that total and ionized calcium levels were comparable between groups. The maintenance of ionized calcium within the clinical reference range in patients with PIH/PE may result from effective physiological adaptation, such as increased production of 1,25(OH)_2_D_3_ and mobilization of bone reserves. However, this does not negate the hypothesis regarding calcium’s role, as homeostasis may be preserved at the expense of bone health or hormonal mechanisms.

The study indicates that the PIH/PE group is characterized by significantly higher obesity (BMI ≈ 33.0 kg/m^2^), aligning with the global risk profile for hypertensive disorders of pregnancy. Many patients do not adhere to dietary recommendations, resulting in excessive weight gain during pregnancy and subsequent obesity, which often becomes permanent [30]. Additionally, obese patients face a significantly higher risk of developing hypertension and comorbidities such as diabetes. Being overweight or obese reduces circulating levels of the active form of vitamin D due to its accumulation in adipose tissue, which may explain the lower 25(OH)D levels observed in these patients [31–33]. The lack of a significant correlation between BMI and 25(OH)D (*r* ≈  0.04) in this cohort necessitates critical discussion. Although a negative association is epidemiologically expected due to sequestration in adipose tissue, this finding may reflect interventions, such as supplementation, later in pregnancy, which mitigated but did not eliminate the intergroup difference. Consequently, 25(OH)D concentrations in the third trimester may be too variable and compensated to serve as reliable risk predictors in this cross-sectional study.

Furthermore, this observational study assessed the effects of vitamin D and calcium supplementation on serum concentrations in the context of PIH and PE. Ideally, a healthy, balanced diet should suffice to maintain adequate levels of calcium and vitamin D during pregnancy. In this study, supplementation with either calcium or vitamin D was reported by 83.7% of women in the normotensive group compared to only 73.2% in the group with gestational hypertension or PE. Although supplementation appeared more common among normotensive women, the timing, dosage, and type of supplementation were not standardized, limiting interpretability. This notable difference may suggest a potential protective effect of supplementation; however, causality cannot be established due to the observational nature of the study. Future research should focus on the preventive role of early and adequate calcium and vitamin D supplementation, particularly for women with elevated BMI, who may be at higher risk for metabolic disturbances and hypertensive complications.

Our observations of insufficient 25(OH)D status and high obesity levels in the PIH/PE group support the recommendation for individualized supplementation. Patients with obesity (BMI ≈ 33 kg/m^2^) may require higher doses, as suggested by previous trials, but this requires confirmation in early pregnancy randomized controlled trials [27].

The timing of vitamin D supplementation initiation is crucial for preventing hypertension onset. Deficiencies occurring in the first trimester may significantly influence the pathogenesis of PE, given the key processes involved in placental development and spiral artery remodeling [34–36]. The WHO recommends daily calcium supplementation of 1.5–2.0 g for pregnant women in populations with low dietary calcium intake to reduce the risk of PE [37].

Strengths of this study include the use of standardized laboratory assays, multivariable adjustment for confounders (BMI, gestational age, diabetes), and transparent reporting of effect sizes, CIs, and corrected *P* values.

However, this study has several limitations. Testing vitamin D and calcium levels in the third trimester may not accurately reflect the true risk of PE, as the first trimester is critical in this regard. This is particularly problematic when attempting to predict PE risk during the third trimester, where current serum levels may appear normal—likely due to patients commencing vitamin D and calcium supplementation in the second trimester. Samples were collected post-diagnosis; thus, associations cannot be interpreted as predictive or causal. Additionally, seasonality and dietary calcium intake were not measured. Evaluating bone mineralization levels in the third trimester using radiofrequency echographic multispectrometry (REMS) or quantitative ultrasound (QUS) may provide insights into vitamin D levels during the first trimester.

Findings support the hypothesis that vitamin D insufficiency and obesity contribute to the pathophysiology of PIH/PE. However, given the observational design, our data should be interpreted as exploratory. Early pregnancy trials are essential to evaluate vitamin D and calcium supplementation as preventive strategies. Current guidelines recommending adequate supplementation (e.g., 600–1000 mg calcium, 600–2000 IU vitamin D daily, with higher doses for obese women) remain the evidence-based standard.

Finally, monitoring 25-hydroxyvitamin D levels in pregnant women may aid in identifying those at increased risk of gestational hypertension and PE. Further research involving larger cohorts and long-term follow-up is needed to clarify the preventive role of vitamin D and calcium supplementation.

## Conclusion

It is important to emphasize that due to the cross-sectional design of this study, the findings are associative and do not permit causal inference. Women with hypertensive disorders of pregnancy exhibited higher BMI and significantly lower 25(OH)D concentrations compared to normotensive controls, while calcium and 1,25(OH)_2_D_3_ levels did not differ. These cross-sectional findings reinforce the association between metabolic disturbances and PIH/PE but cannot establish causality. Larger, prospective studies are warranted to clarify whether early optimization of vitamin D and calcium can reduce the risk of hypertensive complications.

## Data Availability

The datasets generated and analyzed during the current study are not publicly available due to patient confidentiality and ethical restrictions, but are available from the corresponding author on reasonable request and with appropriate institutional approvals.
